# Improving of 6 weeks of repeated sprint training on the aerobic and anaerobic power of college-age male rugby players

**DOI:** 10.3389/fphys.2025.1620197

**Published:** 2025-07-29

**Authors:** Shuo Wang, Jiakai Tang, Shuning Liu, Huixin Li, Qian Li, Liang Pan, Zezhao Chen, Chang Liu

**Affiliations:** ^1^ China Football College, Beijing Sport University, Beijing, China; ^2^ Sports Coaching College, Beijing Sport University, Beijing, China; ^3^ School of Sport Science, Beijing Sport University, Beijing, China; ^4^ School of Strength and Conditioning, Beijing Sport University, Beijing, China

**Keywords:** rugby performance, repeated sprint training, aerobic power, anaerobic power, high-intensity interval training

## Abstract

**Background:**

Rugby is characterized by the necessity for athletes to engage in high-intensity efforts followed by rapid recovery phases. Effective training methodologies that enhance both aerobic and anaerobic capacities are crucial for peak athletic performance in this sport. Objective: This study aimed to assess the efficacy of a 6-week repeated sprint training (RST) program in enhancing the aerobic and anaerobic power of collegiate male rugby players, compared to high-intensity interval training (HIIT).

**Methods:**

Twenty-eight male collegiate rugby players were randomly assigned to one of two groups: repeated sprint training group (RSTG) or a high-intensity interval training group (HIITG). Aerobic power was assessed using the Yo-Yo IR1 Test and an incremental load gas metabolism test, while anaerobic power was measured through the Wingate Anaerobic Test. Assessments were conducted pre- and post-intervention.

**Results:**

All participants completed the study, and all data were included in the analysis. Mixed repeated measures ANOVA revealed significant main effects of time on the Yo-Yo IR1 test, VO_2max_, VT-VO_2_, VT/VO_2max_, LA_10_, peak power (PP), and mean power (MP), indicating significant improvements in both groups post-intervention compared to baseline. Additionally, the time × group interaction effect was significant for VT-VO_2_ and LA_10_. Further paired samples t-test analysis showed that, compared to the HIIT group (HIITG), the repeated sprint training group (RSTG) demonstrated greater intervention effects on Yo-Yo IR1, VO_2max_, VT-VO_2_, VT/VO_2max_, LA_10_, PP, and MP, with more stable improvements.

**Conclusion:**

The 6-week RST protocol was more effective than HIIT in improving key aerobic and anaerobic capacities in collegiate male rugby players. These findings advocate for the integration of RST into the training schedules of rugby players to optimally enhance performance-related physical attributes.

## 1 Introduction

Rugby is a high-intensity, intermittent sport in which players must repeatedly execute high-intensity actions, with brief recovery periods interspersed (Glaise et al., 2022). The ability to perform repeated maximal or near-maximal sprints with minimal recovery time, known as repeated sprint ability (RSA), is considered essential for achieving success in rugby (Fornasier-Santos et al., 2018). During rugby matches, both the aerobic and anaerobic energy systems significantly contribute to player performance. Players are required to maintain high-intensity efforts throughout the game while also having the capacity for explosive movements (Boraczyński et al., 2020).

In recent years, there has been a growing interest in developing effective training methods to enhance both aerobic and anaerobic performance in team-sport athletes. Repeated sprint training (RST) has emerged as a time-efficient and effective approach for improving these physical qualities (Kaynak et al., 2017). The effectiveness of RST lies in its ability to simultaneously stress both the aerobic and anaerobic energy systems, thereby leading to improvements in both capacities (Beyranvand, 2017). Several studies have demonstrated the positive effects of RST on athletic performance. For instance, Delextrat, Gruet, and Bieuzen (2018) found that RST improved both aerobic fitness and muscle oxygenation during repeated sprint sequences in team sport athletes. Similarly, Sökmen et al. (2018) reported significant improvements in both aerobic and anaerobic power following sprint interval training interventions.

Previous research has primarily focused on the application and effects of repeated sprint training (RST) in sports such as volleyball (Kaynak et al., 2017), basketball (Delextrat et al., 2018), tennis (Fernandez-Fernandez et al., 2012), and football (Beato et al., 2019). With respect to rugby, RST still demonstrates promising potential. Beard et al. (2019) found that even as few as four RST sessions per week could effectively enhance repeated power output in international rugby union players. Furthermore, Suarez-Arrones et al. (2014) reported that combining RST with resistance training was more effective in improving repeated sprint performance and muscular power output than RST alone. In addition, the duration of training interventions is a critical consideration in the design of effective training programs. While some studies suggest that short-term RST interventions can yield positive adaptations (Beard et al., 2019), other researchers argue that longer intervention periods may be required for optimal adaptations (Bouten et al., 2023). In other sports, several studies have also confirmed the effectiveness of a 6-week training duration (Fernandez-Fernandez et al., 2012; Ait Ali Braham et al., 2024; Delextrat et al., 2018).

The physiological adaptations to repeated sprint training (RST) have been well-documented. Cipryan and Gajda (2011) found that aerobic capacity influences the ability to perform repeated anaerobic efforts, highlighting the importance of developing both energy systems. Johnston and Gabbett (2011) also confirmed that repeated sprint ability is closely associated with match performance in rugby league players. In recent years, several studies have explored modifications to traditional RST protocols. For example, Panascì et al. (2023) examined the effects of resisted sprint training, while Ait Ali Braham et al. (2024) investigated the impact of incorporating voluntary hypoventilation into RST sessions. These studies suggest that different RST modalities may offer specific advantages for improving various physical performance indicators.

In addition to RST, high-intensity interval training (HIIT) is also widely applied in rugby training. HIIT is a training method that alternates repeated bouts of high-intensity exercise with recovery periods. It effectively stimulates cardiopulmonary function and energy metabolism systems. Compared to continuous aerobic training, HIIT is more time-efficient and enables athletes to reach the “red zone” (≥90% VO_2max_) within a shorter duration. This facilitates the recruitment of large motor units, increases cardiac output, and induces adaptations such as mitochondrial biogenesis and enhanced oxidative metabolism pathways, ultimately improving endurance and recovery capacity (Buchheit and Laursen, 2013; Nicolò and Girardi, 2016). In recent years, HIIT has been widely employed to improve maximal oxygen uptake, agility, sprint speed, and repeated sprint ability (RSA) in youth athletes and team-sport players (Engel et al., 2018; Stankovic et al., 2023). A recent study by Anjali (2024) found that HIIT interventions significantly enhanced speed, agility, and resting heart rate levels in collegiate female rugby players, ultimately improving overall match performance.

In summary, although numerous studies have reported the effectiveness of repeated sprint training (RST) and high-intensity interval training (HIIT) in enhancing rugby performance, two key issues remain to be further explored. First, while RST has been shown to produce significant training effects in professional rugby players (Hamlin et al., 2017), collegiate athletes often face tight academic schedules and limited training time. Therefore, the optimal intervention duration and training protocol for this specific population remain unclear. Second, although both RST and HIIT are widely applied in rugby to improve physical performance, comparative studies examining their respective effects in collegiate rugby players are lacking. In particular, the specific advantages of each training method across different fitness indicators remain to be clarified.

Accordingly, the present study aims to evaluate the effects of a 6-week RST intervention compared to HIIT on aerobic and anaerobic capacities in male collegiate rugby players. Based on previous research and practical experience, it is hypothesized that 6 weeks of RST will result in more pronounced improvements in both aerobic and anaerobic fitness compared to HIIT in this population.

## 2 Materials and methods

### 2.1 Participants

The sample size of participants (i.e., n = 24) was determined using GPower (version 3.1.9.7; Franz Faul, University of Kiel, Kiel, Germany) by using α err prob = 0.05; 1-β Err Prob = 0.8; effect size f = 0.4; test family = *F* test, and statistical test analysis of variance (ANOVA) repeated measures of within-between interaction (20). Therefore, 28 participants (Table 1) were recruited from the college-age male rugby players of Beijing Sport University (n = 28, age: 21.79 ± 2.41 years, body mass: 88.57 ± 3.66 kg, height: 181.18 ± 3.36 cm, estimated body fat: 11.85% ± 0.96%, position: back). To be included in the study participants had to (i) participate in at least 90% of the training sessions, (ii) have regularly competed during the previous competitive season, and (iii) possess medical clearance. To reduce sample heterogeneity and control for positional differences in physical demands and metabolic profiles, only backline players were included in this study. All field tests were completed outdoors on a grass pitch with players wearing football boots. An informed consent signed by the participant was required prior to participation in the study. This study was approved by the sports science experiment ethics committee of Beijing Sport University with the approval number of (No. 2025017H). The experimental scheme and procedure comply with the latest revision of the declaration of Helsinki.

**TABLE 1:** Baseline characteristics and differences in primary outcome measures among participants.

Group and Statistics	Age (year)	Height (cm)	Mass (kg)	Body fat (%)	Yo-Yo IR1 Test (m)	VO_2max_ (mL/kg/min)	VT-VO_2_ (mL/min)	VT/VO_2max_ (%)	LA10 (%)	Peak Power (W/kg)	Mean Power (W/kg)	Fatigue Index (%)
RSTG	22.50 ± 2.62	181.71 ± 1.98	89 ± 3.59	11.41 ± 1.02	1990 ± 473.08	56.27 ± 4.83	3092.20 ± 265.68	73.27 ± 4.82	12.43 ± 1.60	11.02 ± 0.95	8.64 ± 0.74	62.49 ± 6.38
HIITG	21.07 ± 2.02	180.64 ± 2.27	88.14 ± 3.80	12.28 ± 0.68	1938.57 ± 523.33	57.05 ± 5.97	3279.10 ± 343.81	75.65 ± 8.34	13.15 ± 1.29	10.89 ± 1.14	8.72 ± 1.12	59.46 ± 8.77
T					0.273	−0.381	−1.609	−0.927	−1.305	0.333	−0.226	1.042
*P*					0.787	0.707	0.12	0.364	0.204	0.742	0.823	0.308
Hedges’g					0.1	−0.14	−0.591	−0.34	−0.479	0.122	−0.083	0.383
95%CI					(−0.621, 0.819)	(−0.859, 0.582)	(−1.322, 0.152)	(−1.062, 0.388)	(−1.205, 0.256)	(−0.599, 0.841)	(−0.802, 0.637)	(−0.348, 1.106)

Note: RSTG, repeated sprint training group; HIITG, high-intensity interval training group.

### 2.2 Procedures

**FIGURE 1:**
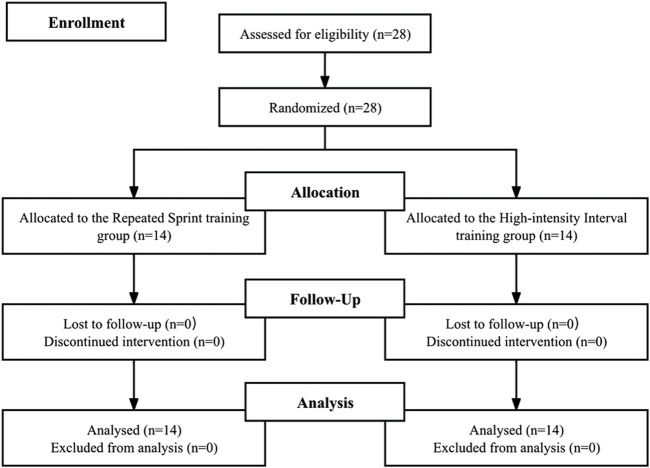
Flow chart of the progress through the phases of the study according to the CONSORT statements.

Pre-tests and post-tests were conducted 1 weeks before and after a 6-week training intervention. All testing sessions were conducted consistently at a fixed time (8:00–11:00 h) and in comparable environmental conditions (20°C–25°C, 30%–35% relative humidity, and an altitude of 87 m). Participants were randomly assigned to either the repeated sprint training group (RSTG; n = 14) or the high-intensity interval training group (HIITG; n = 14) using block randomization in a one-to-one ratio allocation (Figure 1).

### 2.3 Training intervention

During the 6-week training intervention, participants in both groups received two supervised RST or HIIT sessions per week (scheduled on Tuesdays and Fridays). During the remaining time, all participants followed a standardized routine training program, which included two technical and strength training sessions per week (each lasting approximately 1 h). Each training session included a standardized warm-up and cool-down routine. The warm-up (∼15 min) consisted of low-intensity running followed by joint mobility exercises (e.g., shoulder rotations, hip and ankle movements), agility drills, dynamic stretching exercises and a series of 4 progressive 30-m sprints. The cool-down (∼10 min) involved low intensity running and static stretching exercises. Stretching exercises (adductors, quadriceps, hamstrings, calves and Achilles) were held for 15-s and repeated four times with a 15-s rest between each repetition. All stretches were performed below the maximal point of discomfort, avoiding any sensation of pain.

However, the main part of the training session varied based on the training program followed by the participants (Table 2). RSTG’s training involved the repetition of 30-m all-out sprints. Specifically, 6 repetitions were performed, interspersed with 30-s of active recovery, during which the participants jogged back to the starting line. The training volume increased over the intervention period, with 3 series in week 1, 4 series in weeks 2–5 and 5 series in weeks 6–8. The recovery between series was passive and lasted 2 min. The total training time for this specific RSTG segment ranged from 16 to 28 min. The training intensity, volume, mode, time, number of sessions per week and total number of sessions in the RSTG were based on recommendations from previous research (Bishop et al., 2011; Johnston et al., 2014). In contrast, the high-intensity interval training group (HIITG) followed a high-intensity interval training protocol, which consisted of 20-s short running intervals performed at 90% of the individual’s velocity achieved during the final completed stage of the Yo-Yo IR1 Test (VYIR1T) (Coates et al., 2023). The running distance was set according to the standards of the Yo-Yo IR1 Test, and the pace was guided by auditory signals. During week 1, referees performed 2 series of 6 repetitions, increasing to 3 and 4 series in weeks 2–4 and 5–6, respectively. In this training group, a 20-s passive recovery and a 5-min rest were performed between repetitions and series, respectively. The specific training time for this segment ranged from 13 to 31 min. The HIIT protocol was designed to reflect rugby-specific high-intensity running demands and was individualized to each player’s aerobic capacity. Using 90% of the final Yo-Yo IR1 stage velocity, as previously adopted in rugby training research, allowed for a high-intensity but submaximal interval training stimulus. In our study, the corresponding running velocities during HIIT sessions ranged approximately between 13.5 and 15 km/h across participants.

**TABLE 2:** The main part of the training session for repeated sprint training group (RSTG) and high-intensity interval training group (HIITG).

Exercise	The first stage (week 1)	The second stage (week 2–4)	The third stage (week 5–6)
Repeated Sprint training (twice a week)	Intensity: 30-m all-out sprints Volume: 6 rep/series, 3 series Rest: 30 s/rep, 2 min/series	Intensity: 30-m all-out sprints Volume: 6 rep/series, 4 series Rest: 30 s/rep, 2 min/series	Intensity: 30-m all-out sprints Volume: 6 rep/series,5 series Rest: 30 s/rep, 2 min/series
High-intensity interval training (twice a week)	Intensity: 90% V_YIR1T_ Volume: 20 s*6rep/series, 3 series Rest: 30 s/rep, 2 min/series	Intensity: 90% V_YIR1T_ Volume: 20 s*6rep/series, 4 series Rest: 30 s/rep, 2 min/series	Intensity: 90% V_YIR1T_ Volume: 20 s*6rep/series, 5 series Rest: 30 s/rep, 2 min/series

Note: Sec, second; V_YIR1T_, velocity achieved during the final completed stage at the Yo-Yo IR1 test.

The Polar Team2 System (Polar Electro Oy, Kemple, Finland) was used to monitor the heart rate of each player throughout each training session, with data later extracted from custom-specific software (Polar Team2, Electro Oy, Kemple, Finland), in order to obtain maximum heart rate (HRmax), time spent in each HRmax% zone and Training impulse (TRIMP). TRIMP considers the training duration and intensity at the same time and reflects the comprehensive effect of training on the internal and external load of the athlete’s body, as well as the load of medium and high intensity training. The method to determine the athlete’s TRIMP in the current study is based on the formula proposed by Edwards (1994), where the time in each HRmax% zone is multiplied by the corresponding weighting factor for that zone and the results summated. The HRmax of each player was established using the peak value recorded by the monitoring system during the training.

### 2.4 Measurements

The experiment lasted for 8 weeks. In the first week, participants familiarized themselves with all equipment and testing procedures and completed the baseline tests. The intervention training began in week 2 and lasted for 6 weeks, The post-test was scheduled in week 8, following a 48-h standardized recovery period. To minimize the impact of daily training fatigue on test results, all tests were conducted before the participants’ regular training schedule. Specifically, anthropometric measurements and the Wingate anaerobic test were conducted on day one, the Yo-Yo IR1 test on day two, and the incremental load gas metabolism test on day three.

Previous studies have shown that lack of sleep、caffeine intake, poor diet, and fatigue (Charest and Grandner, 2022; Guest et al., 2021; McKay et al., 2023) can affect training outcomes. Therefore, to minimize the interference of irrelevant variables, all participants were instructed to avoid consuming caffeinated foods and beverages for 6 h before the test, maintain a regular lifestyle, and consume a diet rich in carbohydrates. Participants were also advised to refrain from exercise the day before the test and to finish their last non-caffeinated meal at least 3 h prior to the scheduled test time. All tests and training sessions were conducted in an indoor court with a room temperature of 20°C–25°C.

Height (seca 213, seca GmbH & Co. KG, Hamburg, Germany) and body mass (Tanita MC-780U, Tanita Corp., Tokyo, Japan) were measured when individuals arrived at the laboratory. Additionally, the estimated body fat percentage was determined employing bioelectrical impedance principles with a multi-frequency segmental body composition analyzer (Tanita MC-780U, Tanita Corp., Tokyo, Japan).

#### 2.4.1 Aerobic power test

##### 2.4.1.1 Yo-Yo IR1 test

Given the duration of a rugby league match, the distances covered at low speeds, and the need for rapid recovery following high-intensity efforts, it is expected that well-developed aerobic power plays a crucial role in performance (Johnston et al., 2014). The Yo-Yo intermittent recovery test level 1 (Yo-Yo IR1 Test) effectively assesses athletes’ intermittent recovery and aerobic capacity (Krustrup et al., 2003), and has been recently used in rugby league (Gabbett and Seibold, 2013; Chiwaridzo et al., 2017). The test was conducted according to the protocol outlined in the Guide to the Yo-Yo IR1 Test (https://www.theyoyotest.com/). Participants were required to complete 20-m shuttle runs, followed by a 10-s rest interval, with the pace set to a series of beeps. The running speed progressively increased until the participants reached exhaustion.

##### 2.4.1.2 Increasing load of gas metabolism test

An incremental load test was performed using an incremental load treadmill (H/P Cosmos, Germany). Warm-up exercises were performed for 5–10 min before each test. At the beginning of the test, the starting speed of the treadmill was set at 6 km/h, increasing by 1 km/h per minute, until 16 km/h, at which point the speed remained constant, and the slope increased by 1.5% per minute, until the subject was exhausted. Relevant respiratory parameters such as maximum oxygen uptake (VO_2max_), ventilatory anaerobic threshold (VT-VO_2_), and ventilatory anaerobic threshold as a percentage of VO_2max_ (VT/VO_2max_) were measured using a gas metabolism analyzer (Max I, Physio-Dyne Instrument Corp., New York, United States). Among them, VT was determined according to the following criteria:

In the incremental load test, VT is determined (i) when the ratio of ventilation (VE) to carbon dioxide production (VCO_2_) shows a non-linear increase at the inflection point, and (ii) when the load intensity reaches a certain level, when the ratio of VE to oxygen consumption (VO_2_) increases sharply (Binder et al., 2008). VT is determined independently by two investigators. When they are not consistent, and if the difference between the two selected results is significant, VT needs to be re-determined. If the difference can be ignored, the average value is taken.

##### 2.4.1.3 Test of lactate clearance rate

To analyze the aerobic recovery speed of athletes after increasing load and evaluate their recovery ability, blood samples (20 μL of fingertip blood) were collected at rest (before testing while seated) and at 0, 1, 3, 5, 7, and 10 min immediately after the increasing load test. The EKF Biosen s-line automatic blood lactate analyzer (EKF-diagnostic GmbH, Barleben, Germany) was used to measure blood lactate, and the results were recorded, with the lactate clearance rate calculated using the following formula (Wang et al., 2019):

$${LA}_{10}\%=\frac{{LA}_{\max}\%-{LA}_{10}}{{LA}_{\max}-{LA}_{rest}}$$

where LA_10_ means the lactate clearance rate at 10 min after testing, LAmax represents the peak lactate value after testing, LA_10_ is the lactate value at 10 min after testing, and LArest is the lactate value before testing.

#### 2.4.2 Anaerobic power test

##### 2.4.2.1 Wingate Anaerobic Test

The Wingate Anaerobic Test (WAnT) is used to assess athletes’ anaerobic power and capacity. This test has recently been widely used in rugby league players’ testing (Pienaar and Ben, 2013) as an important tool for evaluating training effects. The test was conducted as per the method described by Bar-Or (1987). Peak power (PP), mean power (MP), and fatigue index (FI) were used as indicators to assess the anaerobic power of participants. A 30-s all-out Wingate test was performed on a Monark cycloergometer (Ergomedic 828E, Vansbro, Sweden). Before the test, a warm-up protocol was conducted consisting of 5 min of pedaling at low intensity (i.e., subjects chose the load and cadence), followed by another 5 min of pedaling at 60 revolutions per minute (rpm) with a load of 2 kiloponds (Kp). During the last 5 s of each minute, the subjects performed a maximal intensity sprint. After 2 minutes of recovery, the Wingate test began. Subjects pedaled as fast as possible for 30 s against a constant load (Kp) calculated according to 7.5% of each participant’s body mass (Bar-Or, 1987). The instructions given to them were: i) reach maximal rpm in the shortest time and ii) try to maintain the highest number of rpm until the end of the test. During the test, subjects were encouraged by 4 researchers from start to finish. The peak power (PP), mean power (MP), and fatigue index (FI) of each participant were then calculated based on the results of the test.

### 2.5 Statistical analysis

The primary outcome measures of this study (Yo-Yo IR1, VO_2max_, VT-VO_2_, VT/VO_2max_, LA_10_, PP, MP, and FI) were analyzed using SPSS software (version 28.0). Data are presented as mean ± standard deviation (M ± SD). The normality of the data was assessed using the Shapiro-Wilk test, and homogeneity of variance was verified with Levene’s test. Mauchly’s test of sphericity was conducted to examine the assumption of sphericity; if violated, the Greenhouse-Geisser correction was applied.

To ensure baseline comparability between groups, independent samples t-tests were conducted to compare the pre-intervention values of the two groups. The same test was also used to examine between-group differences in post-intervention outcomes. A mixed-design repeated measures ANOVA was performed to evaluate the effects of group (HIITG vs. RSTG), time (pre-vs. post-intervention), and their interaction. When a significant interaction effect was detected, simple effects analysis was conducted to identify the specific source of the significant differences, and the results were visualized using interaction plots. For variables showing main or interaction effects, paired samples t-tests were used to examine within-group pre–post differences.

All results were reported with 95% confidence intervals (95% CI) for effect sizes. For mixed-design repeated measures ANOVA, partial eta squared (partial η^2^) was reported, with interpretation thresholds based on Ferguson (2009): no effect (<0.04), small (0.04–0.24), medium (0.25–0.63), and large (≥0.64). For all other analyses, Cohen (2013) was used to estimate effect sizes, with interpretation thresholds (absolute values) based on Smith et al. (2016): trivial (<0.2), small (0.2–0.6), moderate (0.6–1.2), large (1.2–2.0), and very large (>2.0). When the sample size per group was less than 20, Hedges’ g correction was applied for effect size and confidence interval estimation. The significance level for all statistical models was set at ^*^ *P* < 0.05, with ^**^ *P* < 0.01 considered highly significant.

Formulas for the calculation of these effect sizes are detailed in the work of Fritz et al. (2012). In addition, to assess differences in internal training load between the RST and HIIT groups, independent samples t-tests were used to compare the TRIMP values per training session across groups.

## 3 Results

All participants completed the 6-week training intervention without dropout, and their data were included in the final analysis. All data were normally distributed (*P* > 0.05) and satisfied the assumptions of homogeneity of variance and sphericity.

At baseline, there were no significant differences (*P* > 0.05) between the two randomly assigned groups in the pre-test results of Yo-Yo IR1, VO_2max_, VT-VO_2_, VT/VO_2max_, LA_10_, PP, MP, and FI.

In terms of TRIMP (training impulse), the average TRIMP per session was 61.8 ± 8 AU in the RST group and 73.4 ± 9.2 AU in the HIIT group. The total accumulated TRIMP over the 6-week intervention was 741 AU for the RST group and 881 AU for the HIIT group. Independent samples t-test showed a significant difference between the groups (T (22) = 3.321, ^**^ *P* < 0.01, Hedges’ g = 1.309, 95% CI [–2.160, −0.434]) (Table 3), reflecting the higher training density of the HIIT protocol.

**TABLE 3:** Session-by-session TRIS1MP values for RST and HIIT groups during the 6-week intervention period.

Week	Session No.	RST Group TRIMP(AU)	HIIT Group TRIMP(AU)
1	1	50	60
	2	51	62
2	3	52	64
	4	58	66
3	5	59	68
	6	62	73
4	7	64	76
	8	65	78
5	9	66	81
	10	68	83
6	11	72	84
	12	74	86

**FIGURE 2:**
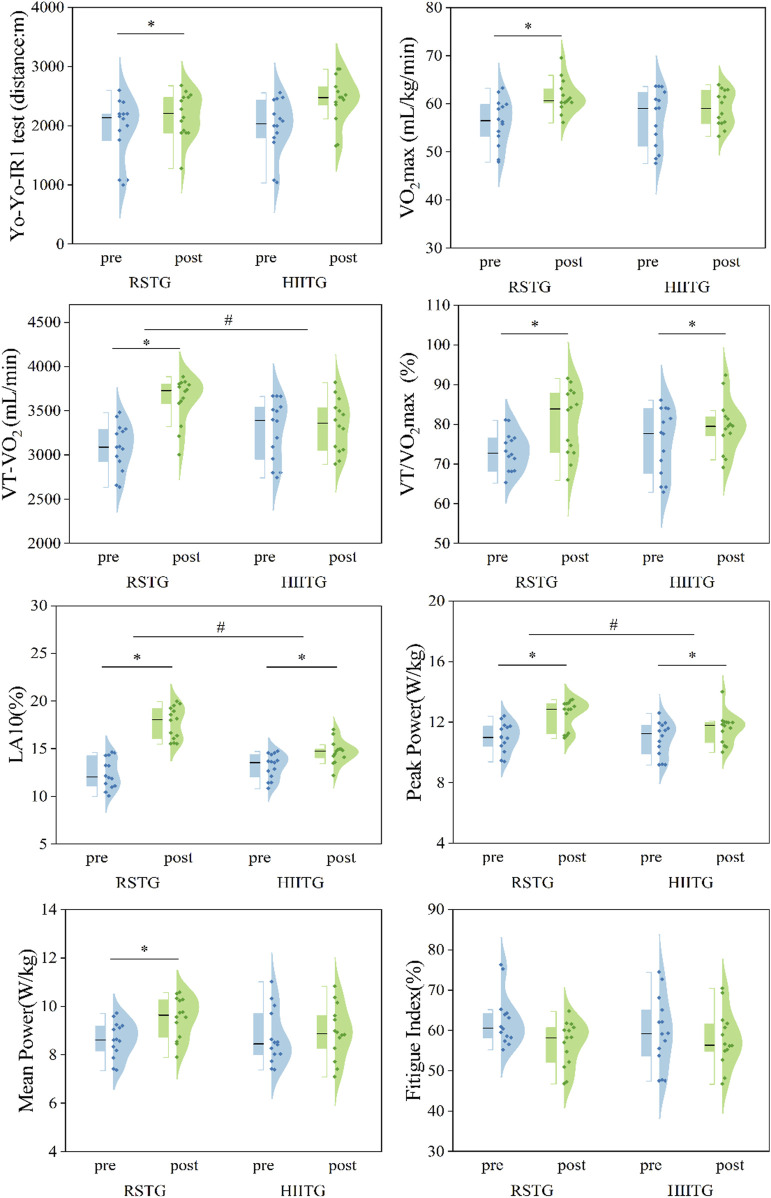
The task performance before and after Training. Each spot on the figure reflected one participant, and the mean of each group was presented. Note: * indicates a significant within-group difference before and after intervention, and # indicates a significant between-group difference after intervention.

**FIGURE 3:**
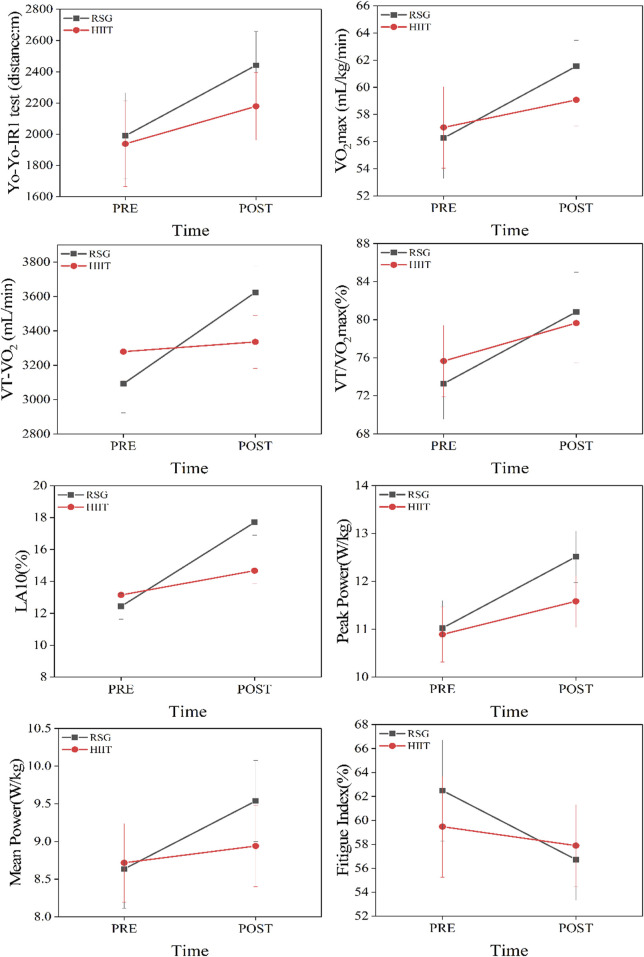
Interaction plots from the mixed-design repeated measures ANOVA.

After 6 weeks of training, all performance indicators except FI showed greater improvements in the RSTG than in the HIITG. Mixed repeated measures ANOVA results (Figures 2, 3) indicated that none of the confidence intervals crossed zero (Tables 4, 5).

**TABLE 4:** The results of the mixed-design repeated measures ANOVA.

Group and Statistics		Yo-Yo IR1 Test (m)	VO_2max_(mL/kg/min)	VT-VO_2_(mL/min)	VT/VO_2max_ (%)	LA10 (%)	Peak Power (W/kg)	Mean Power (W/kg)	Fatigue Index (%)
Time	F	21.929	12.351	108.512	21.569	92.22	52.041	12.734	3.47
	*P*	0*	0.002*	0*	0*	0*	0*	0.001*	0.074
	partial η²	0.458	0.322	0.807	0.453	0.78	0.667	0.329	0.118
	95%CI	(1770.509, 2158.063)	(54.547,58.769)	(3066.303, 3305.001)	(71.814, 77.106)	(12.227, 13.356)	(10.55, 11.364)	(8.307, 9.046)	(54.89, 59.728)
		(2156.86, 2463.14)	(58.922,61.67)	(3371.03, 3588.34)	(77.293, 83.174)	(15.618, 16.759)	(1.667, 12.428)	(8.858, 9.62)	(58, 63.961)
Group	F	1.054	0.373	0.222	0.061	7.421	2.271	0.613	0.281
	*P*	0.314	0.547	0.641	0.806	0.011*	0.144	0.441	0.601
	partial η²	0.039	0.014	0.008	0.002	0.222	0.08	0.023	0.011
	95%CI	(1993.257, 2438.172)	(56.033, 60.072)	(3201.896, 3514.094)	(73.527, 80.566)	(14.451, 15.684)	(11.255, 12.28)	(8.608, 9.565)	(57.056, 62.164)
		(1836.114, 1836.114)	(56.882, 60.921)	(3151.243, 3463.441)	(74.128, 81.166)	(13.296, 14.529)	(10.724, 11.749)	(8.35, 9.307)	(56.125, 61.233)
Time* Group	F	2.05	2.483	70.828	2.066	28.024	6.966	4.639	1.126
	*P*	0.164	0.127	0#	0.163	0#	0.014	0.041	0.298
	partial η²	0.073	0.087	0.731	0.074	0.519	0.211	0.151	0.042
	95%CI	(1715.958, 2264.042)	(53.282, 59.252)	(3110.317, 3447.886)	(71.911, 79.396)	(11.634, 13.231)	(10.447, 11.599)	(8.113, 9.158)	(58.277, 66.707)
		(2224.856, 2658.001)	(59.593, 63.479)	(3181.921, 3489.244)	(75.482, 83.798)	(16.895, 18.509)	(11.974, 13.05)	(8.998, 10.076)	(53.307, 60.149)
		(1664.529, 2212.614)	(54.064, 60.034)	(2923.418, 3260.987)	(69.524, 77.009)	(12.351, 13.949)	(10.315, 11.467)	(8.195, 9.24)	(55.254, 63.684)
		(1961.999, 2395.144)	(57.113, 60.999)	(3470.126, 3777.449)	(76.668, 84.985)	(13.868, 15.481)	(11.044, 12.12)	(8.401, 9.479)	(54.468, 61.31)

Note: *indicates a significant main effect of time or group; ^#^indicates a significant time × group interaction effect.

**TABLE 5:** Paired samples t-test results for within-group pre–post differences and independent samples t-test results for post-intervention between-group differences.

Variable	HIITG (N=14)						RSTG (N=14)						Group (post)			
	Pre	Post	T (13)	*P*	Hedges’g	95%CI	Pre	Post	T	*P*	Hedges’g	95%CI	T (26)	*P*	Hedges’g	95%CI
Yo-Yo IR1 Test (m)	1938.57 ± 523.33	2178.57 ± 385.54	−1.769	0.1	−0.499	(−1.144, 0.146)	1990 ± 473.08	2441.43 ± 402.72	−7.741	0	−0.95	(−1.433, −0.468)	1.764	0.089	0.647	(−0.99, 1.382)
VO_2max_(mL/kg/min)	57.05 ± 5.97	59.06 ± 3.64	−1.227	0.242	−0.386	(−1.086, 0.313)	56.27 ± 4.83	61.54 ± 3.43	−4.153	0.001	−1.2	(−2.005, −0.395)	1.855	0.075	0.681	(−0.068, 1.417)
VT-VO_2_(mL/min)	3279.10 ± 343.81	3335.58 ± 295.30	−1.264	0.229	−0.165	(−0.455, 0.125)	3092.2 ± 265.68	3623.79 ± 263.19	−15.417	0	−1.951	(−2.822, −1.081)	2.726	0.011	1	(0.224, 1.760)
VT/VO_2max_(%)	75.65 ± 8.34	79.64 ± 6.52	−2.566	0.023	−0.499	(−0.968, −0.029)	73.21 ± 4.82	80.83 ± 8.49	−3.895	0.002	−0.99	(−1.681, −0.299)	0.415	0.682	0.152	(−0.570, 0.871)
LA10 (%)	13.15 ± 1.29	14.67 ± 1.22*	−7.135	0	−1.178	(−1.791, −0.564)	12.43 ± 1.60	17.70 ± 1.68*	−7.813	0	−3.113	(−4.688, −1.538)	5.455	0	2.002	(1.089, 2.889)
Peak Power (W/kg)	10.89 ± 1.14	11.58 ± 1.00*	−2.702	0.018	−0.621	(−1.184, −0.059)	11.02 ± 0.95	12.51 ± 0.96*	−9.257	0	−1.518	(−2.252, −0.784)	2.512	0.019	0.922	(0.153, 1.675)
Mean Power (W/kg)	8.72 ± 1.12	8.94 ± 1.10	−0.53	0.445	−0.195	(−0.736, 0.346)	8.64 ± 0.74	9.54 ± 0.85*	−6.491	0	−1.079	(−1.661, −0.498)	1.611	0.119	0.591	(−0.151, 1.322)
Fatigue Index (%)	59.46 ± 8.77	57.89 ± 6.82	0.588	0.567	0.195	(−0.525, −0.914)	62.49 ± 6.38	56.73 ± 5.57	1.999	0.067	0.936	(−0.151, 2.022)	−0.493	0.626	−0.181	(−0.900, 0.542)

Yo-Yo IR1 test (m): Significant time effect (F (1,26) = 21.929, ^**^ *P* < 0.01, partial η^2^ = 0.458) suggested both groups improved significantly post-intervention. Group effect and interaction were not significant (*P* > 0.05). Independent t-test showed no significant between-group difference post-test (*P* > 0.05). Paired t-test revealed RSTG significantly improved from 1990.00 ± 473.08 m to 2,441.43 ± 402.72 m (T (13) = 7.741, ^**^ *P* < 0.01, Hedges’ g = 0.95, 95% CI [–1.433, −0.468]); confidence interval did not cross zero, indicating stable improvement. HIITG improved from 1938.57 ± 523.33 m to 2,178.57 ± 385.54 m (T (13) = 1.769, ^*^ *P* < 0.05, Hedges’ g = 0.499, 95% CI [–1.144, 0.146]); although significant, CI crossed zero, indicating unstable effect.

VO_2max_ (mL/kg/min): Time effect was significant (F (1,26) = 12.351, ^**^ *P* < 0.01, partial η^2^ = 0.322). No significant group or interaction effects. RSTG improved significantly from 56.27 ± 4.83 to 61.54 ± 3.43 (T (13) = 4.153, ^**^ *P* < 0.01, Hedges’ g = 1.20, 95% CI [–2.005, −0.395]); stable effect. HIITG change from 57.05 ± 5.97 to 59.06 ± 3.64 was not significant (*P* > 0.05).

VT-VO_2_ (mL/min): Time effect significant (F (1,26) = 108.512, ^**^ *P* < 0.01, partial η^2^ = 0.807), and interaction highly significant (F (1,26) = 70.828, ^**^ *P* < 0.01, partial η^2^ = 0.731). Post-test difference was significant (T (26) = 2.726, ^*^ *P* < 0.05, Hedges’ g = 1.000, 95% CI [0.224, 1.760]). RSTG improved from 3092.2 ± 265.68 to 3623.79 ± 263.19 (T (13) = 15.417, ^**^ *P* < 0.01, Hedges’ g = 1.951), while HIITG did not show significant change.

VT/VO_2max_ (%): Time effect significant (F (1,26) = 21.569, ^**^ *P* < 0.01, partial η^2^ = 0.453), no group or interaction effects. RSTG improved from 73.21% ± 4.82% to 80.83% ± 8.49% (T (13) = 3.895, ^**^ *P* < 0.01, Hedges’ g = 0.990, CI does not cross 0). HIITG improved from 75.65% ± 8.34% to 79.64% ± 6.52% (T (13) = 2.566, ^*^ *P* < 0.05, Hedges’ g = 0.499), both showing stable improvements.

LA_10_ (%): Time effect (F (1,26) = 92.220, ^**^ *P* < 0.01, η^2^ = 0.780), group effect (F (1,26) = 7.421, ^*^ *P* < 0.05, η^2^ = 0.222), and interaction effect (F (1,26) = 28.024, ^**^ *P* < 0.01, η^2^ = 0.519) were all significant. RSTG improved from 12.43% ± 1.60% to 17.70% ± 1.68% (T (13) = 7.813, ^**^ *P* < 0.01, Hedges’ g = 3.113), HIITG from 13.15% ± 1.29% to 14.67% ± 1.22% (T (13) = 7.135, ^**^ *P* < 0.01, Hedges’ g = 1.178); RSTG had greater effect.

Peak Power (W/kg): Time effect (F (1,26) = 52.041, ^**^ *P* < 0.01), interaction effect (F (1,26) = 6.966, ^*^ *P* < 0.05). RSTG improved from 11.02 ± 0.95 to 12.51 ± 0.96 (T (13) = 9.257, ^**^ *P* < 0.01, Hedges’ g = 1.518). HIITG from 10.89 ± 1.14 to 11.58 ± 1.00 (T (13) = 2.702, ^*^ *P* < 0.05, Hedges’ g = 0.621); both stable, RSTG greater.

Mean Power (W/kg): Time effect significant (F (1,26) = 12.734, ^**^ *P* < 0.01, partial η^2^ = 0.329), interaction effect significant (F (1,26) = 4.639, ^*^ *P* < 0.05). RSTG improved from 8.64 ± 0.74 to 9.54 ± 0.85 (T (13) = 6.491, ^**^ *P* < 0.01, Hedges’ g = 1.079), stable effect. HIITG not significant.

Fatigue Index (%): No significant time, group, or interaction effects (*P* > 0.05). Independent and paired t-tests showed no significant changes in either group, indicating that neither intervention significantly affected FI.

## 4 Discussion

The primary aim of this study was to compare the effects of a 6-week repeated sprint training (RST) and high-intensity interval training (HIIT) intervention on the aerobic and anaerobic power of college-age male rugby players. The results of the study indicate that both training methods effectively improved specific fitness parameters in this population. However, over the 6-week intervention period, RST proved to be more advantageous than HIIT in enhancing both aerobic and anaerobic performance, particularly in terms of ventilatory threshold (VT), lactate clearance rate, and power output.

The RSTG improved athletes’ aerobic capacity, as evidenced by increases in VO_2max_ and Yo-Yo IR1 test performance. Consistent with the findings of Boer and van Aswegen (2016) and Maggioni et al. (2019), the present study further confirms the effectiveness of RST in enhancing aerobic capacity and the efficiency of energy systems in high-intensity intermittent sports. The superior improvements observed in VO_2max_, ventilatory threshold (VT), and Yo-Yo IR1 scores in the RST group can be attributed to the higher training intensity compared to HIIT. Although both groups had similar rest intervals, the greater intensity of RST provided a stronger cardiovascular stimulus, thereby promoting more pronounced aerobic adaptations. This high-intensity training may have contributed to increased capillary density, mitochondrial biogenesis, and elevated oxidative enzyme activity (Serpiello et al., 2012), all of which are crucial for improving aerobic endurance. The high metabolic load may also have enhanced stroke volume and cardiac output, further boosting aerobic capacity (Crisafulli et al., 2011).

In the context of anaerobic power, both RST and HIIT led to increased peak and mean power output, as reflected in the results of the Wingate Anaerobic Test. The improvements in the RSTG align with previous studies, such as Vasconcelos et al. (2020) and Gutknecht et al. (2022), which have highlighted the efficacy of repeated sprint protocols in enhancing anaerobic power. Compared to the HIITG, the greater improvements in anaerobic metrics (PP and MP) in the RSTG can be explained by the higher intensity of the RST sessions, which placed a greater emphasis on the glycolytic energy system and led to enhanced recruitment of fast-twitch muscle fibers (Mendez-Villanueva et al., 2008). The short, intense bursts during RST primarily engage type II muscle fibers, enhancing their contractile properties and increasing glycolytic enzyme activity, which contributes to improved anaerobic power (Girard et al., 2011). The progressive increase in training volume in the RSTG also likely provided an additional stimulus for anaerobic adaptations, such as increased phosphocreatine resynthesis and enhanced buffering capacity. Although the HIITG also showed improvements in VO_2max_, ventilatory threshold, and peak power, the magnitude of improvement was smaller than that of the RSTG, and the effects were less consistent. This finding aligns with the mechanism by which HIIT stimulates cardiovascular and metabolic adaptations through high-intensity loads (Messonnier et al., 2013).

One key result from our study was the significantly enhanced lactate clearance ability (LA_10_) in the RSTG compared to the HIITG. This improvement is consistent with the findings of Iaia et al. (2017), who also observed enhanced lactate clearance following RST. Compared to HIIT, the higher intensity sprints in RST likely provided a greater stimulus for adaptations in lactate transporters (e.g., monocarboxylate transporters) and oxidative enzyme activity, leading to a more efficient management of metabolic by-products (Ulupınar et al., 2024). The higher intensity facilitated an upregulation of lactate dehydrogenase and other enzymes involved in the Cori cycle, which helped in more efficient lactate clearance and conversion back to usable energy (Messonnier et al., 2013). The similar rest periods between RST and HIIT may explain why improvements in LA_10_ were primarily driven by the higher intensity of the sprints rather than differences in recovery.

Interestingly, our study found no significant changes in the fatigue index (FI) for either group, which contrasts with the findings of Takei et al. (2020), who reported reductions in fatigue following sprint-based training interventions. One possible explanation for this discrepancy is that the Wingate test, which is a single maximal effort, may not effectively reflect improvements in fatigue resistance induced by interval-based training like RST and HIIT. Both RST and HIIT involve repeated bouts of exercise with rest periods, which are more likely to enhance recovery capacity rather than the ability to resist fatigue during a single, maximal effort. Consequently, the lack of significant changes in FI may suggest that while these training methods improve recovery during repeated sprints or high-intensity intervals, they do not necessarily lead to enhanced resistance to fatigue in a single maximal effort, such as in the Wingate test. Therefore, the difference in training modality and test characteristics might explain the absence of FI improvement in our study.

Notably, the significantly higher TRIMP scores observed in the HIIT group compared to the RST group reflect the greater cumulative training load and density of the HIIT protocol. This difference in internal load should be considered when interpreting the comparative physiological adaptations between the two training interventions.

## 5 Limitations

Despite the meaningful findings of this study, several limitations should be considered:

First, the sample size was relatively small (n = 14 per group), and all participants were recruited from the same university. This may have limited the statistical power to detect subtle effects and increased the risk of Type II errors. Moreover, the homogeneity of the sample may restrict the external validity of the findings, limiting their generalizability to the broader population of male rugby players. Future studies should consider expanding the sample size to enhance both the stability and external validity of the results.

Second, all participants in this study were male rugby players in backline positions. This restricts the applicability of the findings to other playing positions (e.g., forwards or front-row players), as well as to female rugby athletes. Given the significant differences in physiological characteristics and performance demands across positions, and the biological differences between male and female athletes, the observed training adaptations may not be generalizable to these groups. Future research should incorporate stratified analyses based on sex and playing position.

Third, the intervention period was relatively short—only 6 weeks. Although significant short-term improvements were observed and were consistent with previous findings (Beard et al., 2019; Glaise et al., 2024), it remains unclear whether these training effects are sustainable. Longitudinal follow-up studies are needed to assess the durability and long-term retention of the training-induced benefits.

Fourth, although the Wingate anaerobic test is widely used to evaluate anaerobic capacity, it is a single maximal effort test and may not adequately reflect the improvements in anaerobic performance induced by intermittent training programs such as RST and HIIT (Sökmen et al., 2018). Furthermore, while laboratory indicators such as VO_2max_, VT, and power output provide valuable physiological data, they may not fully capture how these adaptations translate into actual match performance. Future studies should consider using performance assessments with greater ecological validity, such as repeated sprint tests or the Bronco test (Sella et al., 2021), which are widely utilized in rugby training (Johnston and Gabbett, 2011), to comprehensively evaluate training outcomes.

Fifth, although a 48-h recovery period was provided before post-testing, this may have been insufficient for a progressive, high-intensity training schedule and may have resulted in functional overreaching, thereby affecting the outcome measures. Future studies should consider lengthening or individualizing the recovery period to more accurately reflect the true level of training adaptation.

Lastly, although participants were instructed to maintain their usual lifestyle habits, variables such as dietary intake, sleep quality and duration, and daily physical activity were not strictly monitored. These uncontrolled variables may have influenced training adaptation and physiological responses (Charest Grandner, 2022; Guest et al., 2021; McKay et al., 2023). For example, inadequate sleep may impair recovery and attenuate training effects, while changes in macronutrient intake (particularly carbohydrate and protein) can influence VO_2max_ and lactate clearance. Additionally, unmonitored physical activity outside of the intervention could increase training load or contribute to fatigue, thereby affecting the results. Future studies should improve monitoring and control of lifestyle variables to more accurately assess the true effects of training interventions.

## 6 Conclusion

In summary, the findings of this study provide strong evidence that a 6-week repeated sprint training (RST) program offers significant advantages in enhancing both aerobic and anaerobic capacity in college-aged male rugby players, surpassing the improvements achieved through high-intensity interval training (HIIT). Under the specific conditions of this study, RST demonstrated more pronounced effects in improving ventilatory threshold (VT) and lactate clearance capacity. Moreover, RST showed greater effect sizes and more stable improvements in Yo-Yo test performance, maximal oxygen uptake (VO_2max_), peak power (PP), and mean power, indicating its robust training benefits. These results highlight the practical value of RST as an effective training strategy.

Furthermore, the significant improvements observed in lactate clearance capacity and ventilatory threshold (VT) in the RST group further indicate that RST promotes superior metabolic adaptations and recovery capacity compared to HIIT. This finding is particularly important, as effective lactate clearance and a higher VT are critical for maintaining competitive performance in sports like rugby, which involve frequent bouts of high-intensity activity.

Despite the positive outcomes associated with both training interventions, the absence of significant improvements in fatigue index (FI) points to the unique demands of maximal effort tests such as the Wingate test, which may not fully capture the endurance improvements gained from intermittent training.

Future research should explore the impact of longer training durations, incorporate a more diverse athlete population, and investigate the long-term retention of training effects. In summary, these findings advocate for the inclusion of RST in training programs aimed at enhancing rugby-specific physical qualities, particularly for collegiate athletes seeking to improve their performance in high-intensity sports.

## Data Availability

The datasets presented in this study can be found in online repositories. The names of the repository/repositories and accession number(s) can be found in the article/supplementary material.
